# A Chinese Prescription Chuanxiong Chatiao San for Migraine: A Systematic Review and Meta-Analysis of Randomized Controlled Trials

**DOI:** 10.1155/2019/2301680

**Published:** 2019-07-31

**Authors:** Yu Wang, Yajun Shi, Xiaofei Zhang, Junbo Zou, Yulin Liang, Jia Tai, Mei Wang, Chunli Cui, Dongyan Guo

**Affiliations:** ^1^Department of Pharmaceutics, College of Pharmacy, Shaanxi University of Chinese Medicine, Xianyang, Shaanxi, China; ^2^Key Laboratory of Basic and New Drug Research of Traditional Chinese Medicine, Shaanxi University of Chinese Medicine, Xianyang, Shaanxi, China

## Abstract

**Backgrounds:**

Chuanxiong Chatiao san (CXCT) is a famous Chinese classical prescription. It has a favorable effect in treating migraine. It is reported that CXCT combined with Western conventional medicine (WCM) could increase the therapeutic efficacy on migraine. The purpose of this paper is to systematically assess the clinical efficacy, safety, and some indexes of CXCT for migraine.

**Methods:**

PubMed, Embase Database, China National Knowledge Infrastructure (CNKI), Wanfang Database, the Cochrane Library, and the CBM were searched from January 2000 to February 2019. We made a detailed record of outcome measurements. Meta-analysis was performed using RevMan 5.3 software.

**Results:**

A total of 3307 patients were included in the 37 articles. Meta-analysis showed that CXCT significantly increased the total efficiency rate (TER), compared with Western medicine treatment (WMC) (P < 0.00001). When CXCT is combined with WMC, the result showed that P < 0.00001. CXCT was significantly reduced the adverse events (AEs) compared with WMC (P < 0.00001). The levels of VAS, number of migraine episodes (NE), and time of headache duration (TD) were significantly reduced (P < 0.00001). Platelet function and blood rheology level were improved via a significantly decrease in 5-HT and *β*-EP (P < 0.00001). Other indicators such as substance P, CGRP high-cut viscosity, low-cut viscosity, plasma viscosity, and fibrinogen were significantly reduced (P < 0.00001).

**Conclusion:**

Our findings provide evidence that CXCT and CXCT combined with WMC have higher efficacy in the treatment of migraine compared with WCM alone. Methodological quality was generally low, so the conclusion of this paper has some limitations and it has to be carefully evaluated.

## 1. Background

Migraine is a common disease that occurs repeatedly. It belongs to one kind of neurovascular disease. There is a pulsating pain on one side of the head when a migraine attack, which is often accompanied by nausea, vomiting and blurred vision. Besides, sound and light stimulation will increase the pain [1, 2]. According to incomplete statistics, 3.3% to 32.6% of women and 0.7% to 16.1% of men are affected each year. In 2001, the World Health Organization (WHO) had listed severe migraine, paralysis of limbs, mental disorders and dementia as the most serious chronic dysfunction diseases [3]. Recurrence and stubbornness of migraine have caused serious problems in the normal life of patients, whereas there currently are the limitations of conventional therapies available.

Western medicine often uses painkillers to treat migraine, such as nonspecific analgesics (nonsteroidal anti-inflammatory drugs (NSAID), sedatives, opioids, etc.) [4]. The effective treatment of migraine is limited by the availability, cost and AEs caused by overusing analgesics and antimigraine drugs of conventional Western medicine [5]. It was reported AEs that included gastrointestinal reactions, neurotoxicity and hepatotoxicity, and addiction. Thus, many migraine patients resort to complementary and alternative medicine [6]. So it is significant to find new drugs to treat migraine.

Recently, clinical trials and systematic reviews of increasing numbers showed that, as compared to WCM therapy, Chinese herbal formula has obvious advantages in the prevention and treatment of this disease [7]. Migraine belongs to the category of “intermittent headache”, “facial attack hby wind” in traditional Chinese medicine. CXCT is from “Prescriptions People's Welfare Pharmacy” and it has a history of more than 1000 years for treating migraine. The CXCT consists of eight herbs,* Ligusticum chuanxiong hort*,* Angelicae Dahuricae Radix, Notopterygium incisum Ting ex H. T. Chang, Asarum sieboldii Miq, Radix Saposhnikoviae Divaricatae, Nepeta cataria L, Mentha haplocalyx Briq, *and* Glycyrrhiza uralensis Fisch* [8]. Until now, CXCT has been widely applied in the clinical treatment of various headaches and it has very well curative for migraine treatment to reduce the frequency and severity of migraine attacks (Level A) [9–11]. The effectiveness of the main components in CXCT has been proven [12]. CXCT can be used to treat various headaches such as migraine as well as some diseases just like dizziness, cold cough, head pain, and rhinitis [13]. The results of clinical trial indicated that CXCT reduced headache duration and intensity of pain; besides, it could reduce the times of migraine headache and improve quality of life in migraine patients. At the same time, it can significantly inhibit platelet aggregation, improve activation, and release response of platelet and decreases the corresponding indexes of hemorheology [14–16]. By inhibiting the contraction of the vascular smooth muscle, CXCT can enlarge the blood flow in the blood vessel to decrease thrombosis, which has certain improvement on brain damage and is beneficial to treat the vascular headache [17]. Therefore, CXCT has a good prospect in the treatment of migraine.

It is crucial to renew the search and appraise to provide the best evidence for migraine. This paper aims to use the principle of evidence-based medicine to conduct meta-analysis and evaluation of clinical efficacy on CXCT for migraine, so as to provide the reference for clinical research and application.

## 2. Methods

### 2.1. Search Strategies

We searched PubMed, Embase Database, China National Knowledge Infrastructure (CNKI), Wanfang Database, the Cochrane Library, and the CBM (documents collected from January 2000 to February 2019) on the treatment of migraine with Chuanxiong Chatiao san. The language of the text is limited to Chinese and English.

Theme for database searching was “Chuanxiong Chatiao san” [Title or Keywords], “Chuanxiong Chatiao san” AND “Migraine” [Title and Keywords]. Supplement articles were obtained by manual retrieval the references in the article.

And relevant RCTs were downloaded into Endnote software (version X8, Thomson Reuters, Inc., New York, USA) for further exploring. We have made detailed records and analysis of relevant data. Duplicate records were removed. The full-text review was performed, while the title/abstract was thought to be thematic.

### 2.2. Inclusion and Exclusion Criteria

The literature included meets the following criteria:

(1) Migraine patients were diagnosed according to the following criteria: International Headache Society (IHS), The Clinical Guidelines for the New Chinese Medicine (TCGNCM), Clinical Diagnosis Symptoms of Study (CDSS), Standard of TCM Diagnosis and Efficacy (STCMDE), Practical Neurology (PN), Diagnostic Criteria and Treatment Points of Internal Medical Diseases (DCTPIMC), Handbook of Clinical Neurology (HCN), Internal Medicine of Traditional Chinese Medicine (IMTCM), or Practical Internal Medicine (PIM). (2) All the substudies were RCTs. (3) In the study A, the experimental group used CXCT Chinese medicine decoction alone and the control group used Western medicine alone or in combination with other drugs. (4) In the study B, the experimental group used CXCT combined with WMC and he control group was treated Western medicine alone. (5) Do not limit the age, gender, race, or other basic conditions of patients. (6) The primary outcome measure was the TER. Effective means that the degree of headache is relieved, and the frequency and duration of headache attacks are reduced. The second outcome measures were AEs and some biological indexes.

The exclusion criteria were as follows:

(1) The article was repeatedly published. (2) The type of the article included animal experiment, review, and case report. (3) Besides CXCT and drugs, acupuncture was used in the experiment group. (4) Trails were not RCTs or there were no criteria. (5) This patient was not diagnosed with migraine. (6) The full-text cannot be found.

### 2.3. Data Extraction and Quality Assessment

This meta-analysis used Review Manager 5.3 software to perform quality assessment. It was evaluated from random sequence generation, allocation concealment, blinding of participants and personnel, blinding of outcome assessment, incomplete outcome data, selective reporting, and other biases and divided into three indexes: “high risk,” “unclear risk,” and “low risk.”

Scientific evaluation of the literature is not only beneficial to the improvement of clinical trials, but also has important significance for the merger and analysis of the articles. These data were separately collected and cross-checked by two researchers, and the controversial content or the score of the article was determined by a third person after careful assessment.

Information collected included the author names, year of publication, numbers of cases, methodological characteristics, outcome measures, adverse events, and follow-up records. These data were separately collected and cross-checked by two researchers. The controversial content was determined by a third person after careful assessment.

### 2.4. Data Analysis

Meta-analysis was using RevMan 5.3 software which published on the Cochrane Collaboration. Heterogeneity was assessed by means of I^2^ statistic. Fixed-effect model of meta-analysis was used to analyze data that were low heterogeneous. I^2^> 50% represented high heterogeneity; random-effect model was used to analyze the data. Outcome measures such as TER and AEs regard as dichotomous variables used the odds ratio (OR) values with 95% confidence interval (95%CI). If outcomes were continuous data, they were presented as weighted mean difference (WMD) and its 95% CI. Funnel plot was used to analyze publication bias.

## 3. Results

### 3.1. Characteristics of the Eligible Studies

**Figure 1:**
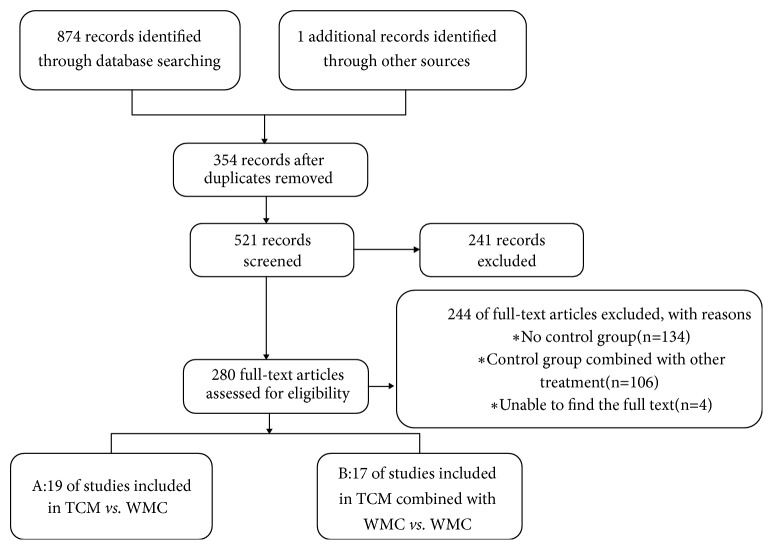
PRISMA 2009 flow diagram (RCT, randomized controlled trial).

A total of 875 articles were retrieved in this study, among which 354 were duplicated. After rescreening the titles and abstracts, 521 articles were included. Finally, 280 articles were excluded after checking the full text. Thus, 36 eligible studies were included. Among them, 19 were compared between CXCT alone and Western medicine alone [14, 17–34], and 17 were CXCT combined with Western medicine compared between Western medicine alone [35–51]. The article screening process is shown in Figure 1.

2017 patients were included in the 19 articles (A). There were 1043 cases in the experimental group and 974 cases in the control group. 1292 patients were included in the 17 articles (B). There were 651 cases in the experimental group and 641 cases in the control group. The analysis of the patient's age and sex in all literature indicated there was no statistically significant difference (P>0.05). The methodology of all trials was RCT. The experimental group of study A was treated with CXCT alone, while the control group was treated with Western medicine alone. The experimental group of study B was treated with CXCT and WMC (CXCT&WMC), while the control group was treated with Western medicine alone. All of included studies used the TER as primary outcome measures and most of included studies used ARs as second outcome measure. Some articles contained indexes of serum 5-HT, *β*-EP, SP levels, indexes of hemorheology and VAS (Tables 1 and 2).

### 3.2. Quality of Included Trials Assessment

According to the Cochrane risk of bias estimation, seven studies used the random number table for grouping [14, 17, 43, 45, 47–49]. One study was randomly allocated according to the visiting sequence [30], the rest of the studies only referred to “randomization”.

One of the included studies mentioned the use of blind method or allocation concealment [43]. Therefore, performance bias and detection bias in other studies are considered to be “unclear risks”. Eleven studies mentioned the follow-up [14, 30, 39, 41, 43–46, 48, 50, 51]. There is no shortage of cases or selective reports, so the attrition bias and reporting bias were assessed as “low risk”.

### 3.3. Outcome Measures with Subgroup Analysis

#### 3.3.1. The Total Efficiency of CXCT* vs.* WMC and CXCT&WMC* vs.* WMC Therapy Alone

**Figure 2:**
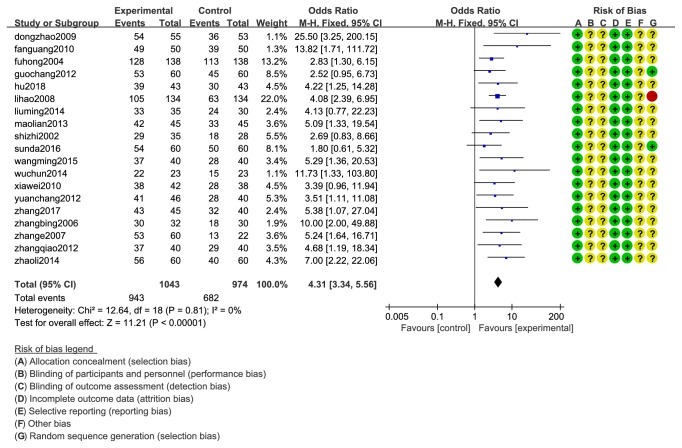
Forest plot of the total effective rate of CXCT and WMC.

The total effective rate was observed in 19 studies and 2017 cases in study A. The total effective rate was 90.41 (943/1043) in the experiment group and 70.02% (682/974) in the control group. Heterogeneity tests of 19 studies showed that there was no heterogeneity (Chi^2^= 12.64; P = 0.81> 0.05; I^2^ = 0%). Fixed-effect model analysis certified that (OR=4.31; 95% CI 3.34 to 5.56; Z=11.21 (P <0.00001)) CXCT was significantly increased the TERs compared with WMC (Figure 2).

**Figure 3:**
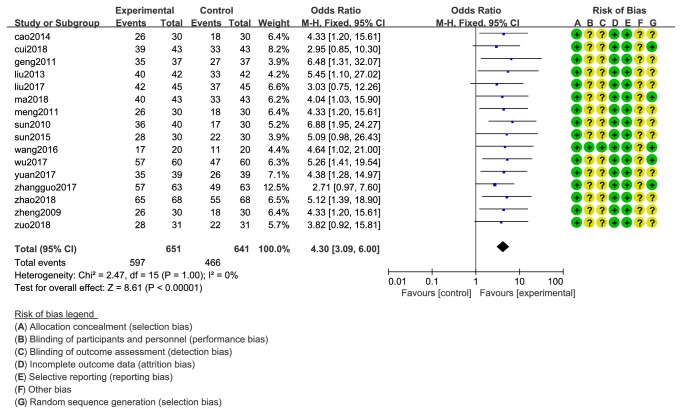
Forest plot of the total effective rate of CXCT&WMC* vs.* WMC.

The total effective rate was observed in 17 studies and 1292 cases in study B. The total effective rate was 91.70% (597/651) in the experiment group and 72.70% (466/641) in the control group. Heterogeneity tests of 17 studies showed that there was no heterogeneity (Chi^2^=2.47; P = 1.00> 0.05; I^2^ = 0%). Fixed-effect model analysis certified that CXCT and WMC (OR=4.30; 95% CI 3.09 to 6.00; Z=8.61 (P <0.00001)) was significantly increased the TERs compared with WMC (Figure 3).

#### 3.3.2. Adverse Events of CXCT* vs.* WMC and CXCT&WMC* vs.* WMC Therapy Alone

**Figure 4:**
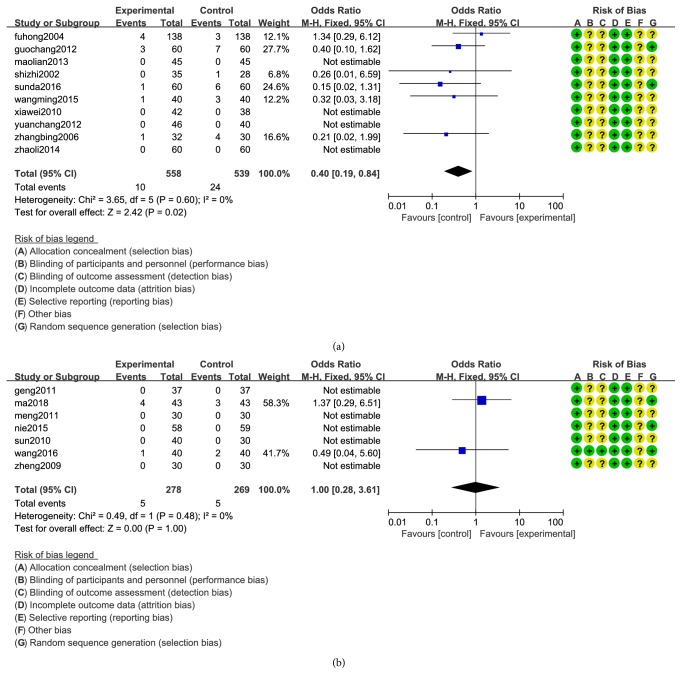
Forest plot of adverse events of medicine.

10 articles mentioned adverse events in study A [14, 17, 19, 20, 23, 25, 26, 32–34], and 7 articles mentioned adverse events in study B [35–38, 41, 43, 49]. With the increasing number of reports on clinical applications, the safety is constantly being examined. The symptoms of AEs occurred in the two groups were: mild nausea, diarrhea, fatigue and lethargy. In the study A, the rate of adverse events was 1.70% (10/588) in the experiment group and 4.22% (24/569) in the control group. Heterogeneity tests showed that there was no heterogeneity (Chi^2^=3.65; P=0.60>0.05; I^2^=0%). Fixed-effect model analysis certified that (OR=0.40; 95% CI 0.19 to 0.84; Z=2.42 (P=0.02)) CXCT was significantly reduced the AEs compared with WMC (Figure 4). In the study B, the data were limited and there was no significant difference in adverse reactions. By comparing group A with group B, it can be concluded that CXCT improves the adverse reactions of WMC and reduces the adverse reactions of WMC.

#### 3.3.3. Ordinary Indexes of CXCT and CXCT&WMC* vs*. WMC Therapy Alone

**Figure 5:**
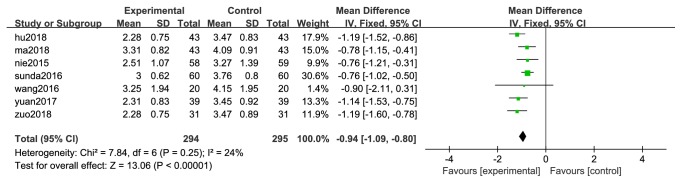
Forest plot of VAS of CXCT&WMC vs. WMC.

**Figure 6:**
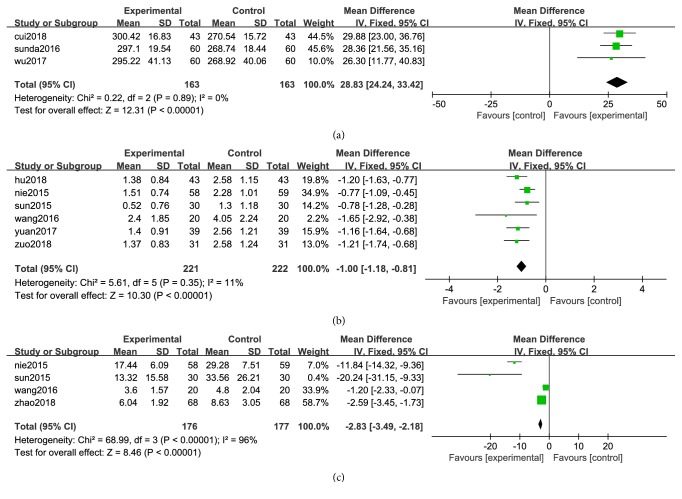
Forest plot of 5-HT (a), NE (b), and TD (c) of CXCT&WMC vs. WMC.

In this paper, some indexes of CXCT for migraine reported in the two studies. VAS is known as the visual analog scoring method for pain assessment and is widely used in China. The lower the VAS score, the better. Seven trails of the two studies evaluated the VAS indicator [14, 18, 41, 43, 46, 49, 51]. A fixed-effect analysis certified that CXCT and CXCT combined with WMC significantly reduced the level of VAS compared to WMC alone (WMD=-0.94; 95%CI -1.09 to -0.80,* P *< 0.00001; Figure 5). The experimental group and the control group were counted on the number of migraine episodes (NE) before and after treatment. The WMD with 95%CI for NE was (WMD =-1.00, 95%CI: -1.18, -0.81) certifying a significant increase in the experimental group compared to control group (P < 0.00001; Figure 6).4 trail [41–43, 50] reported the time of headache duration (TD). The WMD with 95%CI for TD was (WMD=-2.83, 95%CI:-3.49,-2.18), indicating a significant decrease in the headache time in the experimental group compared to control group (P < 0.00001; Figure 6).

#### 3.3.4. Blood Parameters Indexes of CXCT and CXCT&WMC* vs.* WMC Therapy Alone

3 [14, 45, 48] trails provided measures of 5-HT in serum state. There has no heterogeneity in serum (P < 0.00001, I^2^= 0%), a fixed-effect model was thus used for analysis. The WMD and 95% CI for 5-HT was (WMD = 28.83, 95%CI: 24.24, 33.42), indicating a significant increase of 5-HT in experimental group (P = 0.89; Figure 6).

The blood parameters levels of *β*-EP, SP indexes were measured in two trails [14, 48], CGRP was provided in two trails [43, 45]. GMP-140 and TXB2 levels were provided in another trail [23, 52]. The WMD with 95%CI for *β*-EP, SP, CGRP, GMP-140 and TXB2 were (WMD=10.16; 95%CI 8.56 to 11.76), (WMD=-8.84; 95% CI -10.14 to -7.45), (WMD=-2.31; 95%CI -4.01 to -0.61), (WMD=-1.22; 95%CI -2.91 to 0.48) and (WMD=-8.36; 95%CI -16.80 to 0.08). The results showed that the levels of *β*-EP were significantly increased in the experiment group in treating migraine. Besides, SP, CGRP, GMP-140, and TXB2 indexes were significantly decreased in the experiment group.

One study [23] was reported in the indexes of hemorheology, including high shear viscosity, low shear viscosity, plasma viscosity and fibrin glue. Meta-analysis showed significantly decrease in high shear viscosity (WMD=-2.31; 95%CI -2.55 to -2.07) or low shear viscosity (WMD=-0.73; 95%CI -0.82 to -0.64) or plasma viscosity (WMD=-3.91;95%CI-4.33 to -3.49) or fibrin glue (WMD=-1.43; 95%CI -1.89 to -0.97) [23]. The results showed that the indexes of blood viscosity in treating migraine with CXCT are lower than that of WMC. However, the number of included studies was small, so the result of the analysis should be cautious (Table 3).

### 3.4. Bias and Funnel Chart

**Figure 7:**
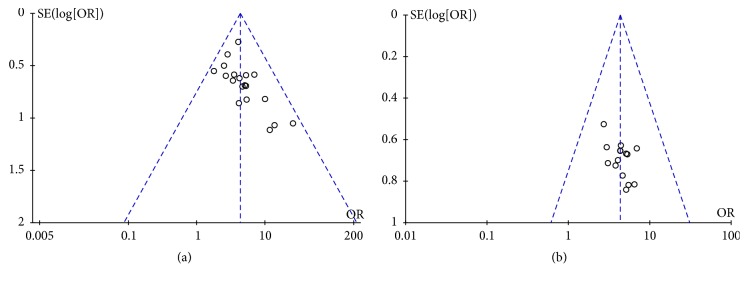
Funnel plot of this study to evaluation the publication bias.

Funnel plot can be used to directly observe the publication bias. The scattered points in the figure are basically symmetric, and there is no obvious publication bias (Figure 7).

## 4. Discussion

Migraine, which is also called by vasodilator headache, is a kind of familial hereditary disease. The mechanism of the disease is not entirely clear, and experts believe that it may be related to heredity, nerve, blood vessels, and others [53]. At present, there are mainly the following theories: vascular theory, neural theory, and trigeminal vascular theory. And the vasculogenic theory was first proposed. With the progress of the time, trigeminal nerve theory has been widely accepted. It was reported that migraine attacks are due to defects in the regulation of pain for trigeminal vascular system [54]. When the brain is stimulated by pain, substances of released regulatory make the cerebral blood vessels dilate, which causes migraine.

The theory of Chinese medicine believes that “Unsmooth will lead to pain”; therefore, Chinese medicine treatment of migraine is mainly improved blood circulation and pain relief. Modern pharmacological research proved migraine and platelet function (GMP-140 and TXB2) were closely related to hemorheology (high shear viscosity, low-cut viscosity, plasma viscosity, and fibrinogen). Since migraine is the result of an inflammatory mechanism mediated by serotonin signaling, leukocyte function, platelet function, and intercellular communication between these cells may be involved in the ultimate pathway of disease [55].

Clinical trials have shown that CXCT can reduce the duration of headaches and reduce the number of headache attacks in migraine patients. The composition of CXCT may affect the expression of CGRP and ET-1 genes which can reduce the synthesis of CGRP and ET-1, then weaken vasomotor of blood vessels which can prevent migraines [16, 17]. There is growing modern pharmacology studies revealed that* Ligusticum chuanxiong hort* has favorable pharmacological activity and effect. Main effective ingredients of* Ligusticum chuanxiong hort* are senkyunolide and ligustrazine, both of which can reduce calcium ions and other related factors and regulate the formation of NO in plasma and the brain to alleviate migraine. The mechanism of pain relief for migraine model rats may be through adjustment of monoamine neurotransmitter levels and turnover, as well as reduction of nitric oxide levels in the blood and brain. Therefore, senkyunolide I may be developed as a potential treatment for migraine [52, 56–58].* Angelicae Dahuricae Radix* can significantly reduce the content of 5-HT in the brain and blood [34]. According to the research, the total coumarin extract (TCE) of* Angelicae Dahuricae Radix* can enhance the antimigraine activity of ligustrazine by reducing the level of head scratches, plasma calcitonin gene-related peptide, serum nitric oxide, and elevating the level of plasma endothelin (p < 0.05) [59]. Other drugs have been reported to have good adjuvant treatment for migraine. Chinese medicine prescriptions can be adjusted to the different symptoms of different patients; it helps to ensure the drug efficacy and rapid recovery. A total of 3307 patients were included in the 37 articles. In the study A, the meta-analysis showed that CXCT had better clinical efficacy than WMC in treating migraine (OR=4.31; 95% CI 3.34 to 5.56; Z=11.21(P <0.00001)). In the study B, the meta-analysis showed that CXCW also had better clinical efficacy than WMC (OR=4.30; 95% CI 3.09 to 6.00; Z=8.61(P <0.00001)). The results showed that the effect of CXCT or CXCT combined with WMC was better than that of WMC alone. Adverse events reported that the experiment group was 1.70% and the control group was 4.22%. 11 studies showed heterogeneity (Chi^2^ = 3.65; P = 0.60> 0.05; I^2^ = 0%). Fixed-effect model analysis certified that (OR=0.40; 95% CI 0.19 to 0.84; Z=2.42 (P=0.02)) CXCT was significantly reduced the AEs compared with WMC. CXCT has more natural ingredients, less adverse events and higher safety. Visual analog scale (VAS) is widely used in pain assessment. The lower the pain index is, the better it is. The decline of VAS (WMD=-0.94; 95%CI -1.09 to -0.80) in the experiment group was lower than those of the control group. The duration of headache and the number of headache episodes are the most objective indicators of headache. Both CXCT and CXCW can significantly reduce the duration of headaches and the number of headache attacks in patients. The number of headache attacks (NE) (WMD =-1.00, 95%CI: -1.18, -0.81) and the duration of headache decreased significantly (TD) (WMD=-2.83, 95%CI:-3.49,-2.18).

High brain 5-HT levels may represent the characteristics of migraine brain and may also be the result of migraine attacks [60, 61]. In this study, 5-HT and *β*-EP indexes were reported, both of which have analgesic effects and can relieve migraine headaches [62–64]. With the increase of substance P, the pain sensitivity of patients will increase [65]. Here we found that 5-HT (WMD=28.83; 95%CI 24.24 to 33.42) and *β*-EP (WMD=10.16; 95%CI 8.56 to 11.76) indexes of experimental group for treating migraine were significantly higher than control group. The level of substance P (WMD=-8.84; 95% CI -10.14 to -7.45) in the experiment group was significantly lower than that of the control group. The increase of GMP-140 and TXB2 in the blood can cause vasoconstriction and aggravate the pain of migraine patients. The results showed that the indexes of GMP-140 (WMD=-1.22; 95%CI -2.91 to 0.48) and TXB2 (WMD=-8.36; 95%CI -16.80 to 0.08) in the experiment group were lower than those of the control group and had statistically significant differences.

The study suggested that some indexes of hemorheology in patients with migraine would increase significantly, which caused increased platelet viscosity [66]. Large amounts of 5-HT were released and consumed, and finally, the reflex expansion of blood vessels occurred, indirectly leading to migraine attacks. The results showed that the high shear viscosity (WMD=-2.31; 95%CI -2.55 to -2.07), low shear viscosity (WMD=-0.73; 95%CI -0.82 to -0.64), plasma viscosity (WMD=-3.91; 95%CI -4.33 to -3.49), and fibrin glue (WMD=-1.43; 95%CI -1.89 to -0.97) of the experiment group were lower than those of the control group. Here we certified that CXCT could improve by increasing the contents of 5-HT and *β*-EP, decreasing the levels of substance P, GMP-140, TXB2, and indexes of hemorheology.

From the above data, CXCT shows its advantages in the treatment of migraine whether used alone or in combination with WMC. There is no specific description of the blind method in the articles selected in this report, but we think that the article is rigorous, so the method of evaluating the article is judged to be “unclear”. When comparing the curative effect of Western medicine and traditional Chinese medicine, it is difficult to adopt blind method. Because the traditional Chinese medicine mostly uses the decoction, but the Western medicine uses the tablet, in the drug dosage form it is very difficult to use the blind method to implement. For doctors, the use of traditional Chinese medicine needs dialectical treatment; each patient needs a different treatment plan, resulting in the fact that the doctor has no way to make a blind diagnosis. This is the difficulty of using blind method in the comparison of traditional Chinese medicine. But the quality of the articles included in this study was relatively low, and the method description was incomplete, so the result of the analysis would be biased. Although there are some limitations in traditional Chinese medicine due to the lack of basic research, evidence-based practices that may be effective make it an attractive treatment system for many diseases. The accuracy of meta-analysis results depends on the article selection. Therefore, the requirements for future experiments should be multicenter, large sample randomized control, and double-blind allocation hidden experiment. In order to improve the quality of the article, we should report cases of withdrawal, follow-up visits, and so on.

## 5. Conclusion

In conclusion, the clinical efficacy of CXCT and CXCT combined with WMC in the treatment of migraine is better than that of WCM alone. In addition, the incidence of adverse reactions of CXCT in the treatment of migraine was significantly lower than that of WCM. However, there was no significant difference in the incidence of adverse events between the two groups.

CXCT and CXCT combined with WMC treatment of migraine 5-HT and beta-EP index were significantly higher than WMC. The VAS, NE, TD, substance P, CGRP, GMP-140, and TXB2 index of the experimental group were lower than those of the control group. The results showed that the blood viscosity of the experimental group was lower than that of the control group.

After evaluating the quality of articles, it is found that most articles are of low quality, which may lead to some limitations of this study. The findings of present study are insufficient evidence given the lack of high-quality evidence. International methodologies and rigorous RCT can produce better tests for CXCT of migraine. Therefore, in order to evaluate the clinical efficacy of traditional Chinese medicine more scientifically and provide a promising development platform for the development of traditional Chinese medicine, more high-quality articles are needed to provide more reliable sources and data for meta-analysis.

The articles included in this report are published in high-quality journals. The blind method is not described in detail in the article, so the judgment in the methodology is “unclear”.

## Figures and Tables

**Table:** (a) Basic characteristic of included studies

Study	Diagnostic standard	Drug E/C		Case E/C	Sex E/C		Ages E/C	
Hu 2018 [18]	NR	Chuanxiong Chatiao san 86g 200ml/day	Nimodipine tablets 120mg/day	43/43	23/20	22/21	36.83	37.57

Sun 2016 [14]	ICHD-II 2004	Chuanxiong Chatiao san 85g 200ml/day	Flunarizine hydrochloride capsules 10mg/day	60/60	NR	NR	NR	NR

Wang 2015 [19]	ICHD-II∖TCGNCM	Chuanxiong Chatiao san 166g	Flunarizine hydrochloride capsules 10mg/day	40/40	15/25	11/29	33.5	34.30

Zhao 2014 [20]	CDSS	Chuanxiong Chatiao san 105g	Flunarizine hydrochloride capsules 5mg/day	60/60	44/76		42	

Liu 2014 [21]	STCMDE	Chuanxiong Chatiao san 133g 400ml/day	Flunarizine hydrochloride capsules 10mg/day	35/30	10/25	10/20	37.5	37

Wu 2014 [22]	NR	Chuanxiong Chatiao san 109g	Flunarizine hydrochloride capsules 10g/day	23/23	5/18	3/20	48	41

Yuan 2012 [23]	PN ∖TCGNCM	Chuanxiong Chatiao san 68g 300ml/day	Flunarizine hydrochloride capsules 10mg/day	46/40	14/32	10/30	32.58	34.46

Guo 2012 [17]	ICHD-II	Chuanxiong Chatiao san 80g	Flunarizine hydrochloride capsules 10mg/day	60/60	22 /38	27/33	52.5	55

Zhang 2012 [24]	ICHD-II∖STCMDE	Chuanxiong Chatiao san 114g	Flunarizine hydrochloride capsules 10mg/day	40/40	12/28	11/29	36.5	38.1

Mao 2013 [25]	ICHD-II	Chuanxiong Chatiao san 105g	Flunarizine hydrochloride capsules 5mg/day	45/45	24/21	22/23	38.8	38.5

Xia 2010 [26]	DCTPIMC∖ STCMDE	Chuanxiong Chatiao san 75g	Flunarizine hydrochloride capsules 5mg/day	42/38	8/34	9/29	35	38

Fan 2010 [27]	NR	Chuanxiong Chatiao san 149g	Flunarizine hydrochloride capsules 10mg/day	50/50	30/20	33/17	NR	NR

Zhang 2017 [28]	IMTCM	Chuanxiong Chatiao san 108g	Ibuprofen 0.6g/day	45/40	6/34	8/37	35.9	35.5

Dong 2009 [29]	IHS 1988	Chuanxiong Chatiao san 146g	Nimodipine Tablets 120mg/day diazepam 7.5mg/day Gammariza 90mg/day	55/53	15/40	16/37	38.7	38.9

Li 2008 [30]	IHS 1988	Chuanxiong Chatiao san 118g 300ml/day	Pizotifen Tablets 0.5mg/day	134/134	61/73	59/75	34	34.6

Zhan 2007 [31]	IHS 1988 ∖TCGNCM	Chuanxiong Chatiao san 100g	Nimodipine Tablets 60mg/day	60/22	22/38	9/13	38.2	37.5

Zhang 2006 [32]	IHS	Chuanxiong Chatiao san 87g	Flunarizine hydrochloride capsules 10g/day	32/30	5/27	6/24	37.96	36.63

Fu 2004 [33]	IHS 1988	Chuanxiong Chatiao san	Paracetamol, Aspirin, Indometacin, Propranolol	138/138	166 /110		36.4	36.4

Shi 2002 [34]	PIM	Chuanxiong Chatiao san 88g 400ml/day	Flunarizine hydrochloride capsules Nifedipine 30mg/day	35/28	11/24	8/ 20	45.8	46.1

**Table:** (b) Quality of included trials assessment

Study	Random method	Treatment/weeks	Withdrawal	Adverse events		follow-up	Outcome measures
Sun 2016 [14]	random number table	4	None	T:1	C:6	12 weeks	TER,VAS,5-HT,SP,*β*-EP

Hu 2018 [18]	randomized controlled	12	None	NR		NR	TER,VAS,AT NE

Wang 2015 [19]	randomized controlled	2	None	T:1	C:3	NR	TER

Zhao 2014 [20]	randomized controlled	4	None	None		NR	TER

Liu 2014 [21]	randomized controlled	2	None	NR		NR	TER

Wu 2014 [22]	randomized controlled	2	None	NR		NR	TER

Yuan 2012 [23]	randomized controlled	4	None	None		NR	TER,GMP-140,TXB2, Hemorheology

Guo 2012 [17]	random number table	2	None	T:3	C:7	NR	TER

Zhang 2012 [24]	randomized controlled	4	None	NR		NR	TER

Mao 2013 [25]	randomized controlled	4	None	None		NR	TER

Xia 2010 [26]	randomized controlled	3	None	None		NR	TER,ABV

Fan 2010 [27]	randomized controlled	2	None	NR		NR	TER

Zhang 2017 [28]	randomized controlled	2	None	NR		NR	TER

Dong 2009 [29]	randomized controlled	4	None	NR		NR	TER, ABV

Li 2008 [30]	Odd even number distribution	4	None	NR		3 months	TER

Zhan 2007 [31]	randomized controlled	4	None	NR		NR	TER

Zhang 2006 [32]	randomized controlled	3	None	T:1	C:4	NR	TER

Fu 2004 [33]	randomized controlled	4	None	T:4	C:3	NR	TER,TD(integral), SP,DP

Shi 2002 [34]	randomized controlled	4	None	None		NR	TER

E, experiment group; C, control group; NR, not report.

IHS, International Headache Society. TCGNCM, The Clinical Guidelines for the New Chinese Medicine. CDSS, Clinical Diagnosis Symptoms of Study. STCMDE, Standard of TCM Diagnosis and Efficacy. PN, Practical Neurology. DCTPIMC, Diagnostic Criteria and Treatment Points of Internal Medical Diseases. HCN, Handbook of Clinical Neurology. IMTCM, Internal Medicine of Traditional Chinese Medicine. GFDTMC, Guidelines for diagnosis and treatment of migraine in China. HMNDT, Handbook of Modern Neurology Diagnosis and Treatment.

PIM, Practical Internal Medicine. TER, Total Efficacy Rate. ABV, average blood velocity in the brain. AT, analgesic time. HT, heal time. SP, systolic pressure. DP, diastolic pressure. SF, seizure frequency.

NE, number of episodes. ND, number of days of attack.

TD, time of duration.

**Table:** (a) Basic characteristic of included studies

Study	Diagnostic standard	Drug E/C		Case E/C	Sex E/C		Ages E/C	
Zheng2009	HCN∖IMTCM	Chuanxiong Chatiao san 63g 400ml/day +Flunarizine hydrochloride capsules 10mg/day	Flunarizine hydrochloride capsules 10mg/day	30/30	12/18	14/16	34.78	37.57

Sun2010	HMNDT∖IMTCM	Chuanxiong Chatiao san 63g 400ml/day +Flunarizine hydrochloride capsules 10mg/day	Flunarizine hydrochloride capsules 10mg/day	40/30	24/16	16/14	35	34.7

Meng2011	HMNDT∖IMTCM	Chuanxiong Chatiao san 63g 400ml/day +Flunarizine hydrochloride capsules 10mg/day	Flunarizine hydrochloride capsules 10mg/day	30/30	12 /18	14/16	34.75	34.72

Geng2011	IHS 1988	Chuanxiong Chatiao san 120g+ Nimodipine 90mg/day	Nimodipine 90mg/day Oryzanol 60mg/day	37/37	15/22	17/ 20	45.8	34.46

Liu2013	NR	Chuanxiong Chatiao san 400ml+Carbamazepine Tablets 0.2g/day	Carbamazepine Tablets 0.2g/day	42/42	15/27	16/26	63.72	52.64

Cao2014	HMNDT∖IMTCM	Chuanxiong Chatiao san 145g 400ml/day +Flunarizine hydrochloride capsules 10mg/day	Flunarizine hydrochloride capsules 10mg/day	30/30	NR	NR	NR	NR

Nie2015	Neurology/IMTCM	Chuanxiong Chatiao san 100g 400ml/day +Flunarizine hydrochloride capsules 10mg/day	Flunarizine hydrochloride capsules 10mg/day	58/59	16/42	15/44	33.07	33.25

Sun2015	Neurology/TCGNCM	Chuanxiong Chatiao san 135g 400ml/day +Flunarizine hydrochloride capsules 10mg/day	Flunarizine hydrochloride capsules 10mg/day	30/30	8/22	7/23	37.25	38.17

Wang2016	ICHD-III/TCGNCM	Chuanxiong Chatiao san 196g 400ml/day +Flunarizine hydrochloride capsules 10mg/day	Flunarizine hydrochloride capsules 10mg/day	20/20	NR	NR	18-65	

Yuan2017	ICHD-ΙΙ	Chuanxiong Chatiao san 87g 400ml/day+ Nimodipine capsules 120mg/day	Nimodipine capsules 120mg/day	39/39	14/25	16/23	32	35

Zhang2017	TCGNCM	Chuanxiong Chatiao san 129g 500ml/day+ Nimodipine capsules 120mg/day	Naproxen 0.6g/day Flunarizine hydrochloride capsules 10mg/day	63/63	33/30	31/32	48.23	48.18

Liu2017	GFDTMC	Chuanxiong Chatiao san 153g/day +Flunarizine hydrochloride capsules 5mg/day	Flunarizine hydrochloride capsules 5mg/day	45/45	16/29	18/27	48.53	47.57

Wu2017	TCGNCM/ICHD-ΙΙ	Chuanxiong Chatiao san 68g/day +Flunarizine hydrochloride capsules 5mg/day	Flunarizine hydrochloride capsules 5mg/day	60/60	19/41	18/42	34.09	34.52

Ma2018	ICHD-ΙΙ	Chuanxiong Chatiao san 153g 400ml/day +Gabapentin 0.9g/day	Gabapentin 0.9g/day	43/43	27/16	29/14	43.21	43.86

Zuo2018	NR	Chuanxiong Chatiao san 63g 400ml/day +Flunarizine hydrochloride capsules 10mg/day	Flunarizine hydrochloride capsules 10mg/day	31/31	13/18	12/19	35.2	34.8

Cui2018	DCTPIMC	Chuanxiong Chatiao san +Flunarizine hydrochloride capsules 10mg/day	Flunarizine hydrochloride capsules 10mg/day	43/43	40/46		41.23	

Zhao2018	DCTPIMC	Chuanxiong Chatiao san 153g+Flunarizine hydrochloride capsules 5mg/day	Flunarizine hydrochloride capsules 5mg/day	68/68	22/46	23/45	52.09	51.07

**Table:** (b) Quality of included trials assessment

Study	Random method	Treatment/weeks	Withdrawal E/C	Adverse events E/C	follow-up	Outcome measures
Zheng2009	randomized controlled	4	None	None	NR	TER, ABV
Sun2010	randomized controlled	4	None	None	NR	TER, ABV
Meng2011	randomized controlled	4	None	None	NR	TER
Geng2011	randomized controlled	4	None	None	NR	TER
Liu2013	randomized controlled	4	None	NR	6 months	TER
Cao2014	randomized controlled	4	None	NR	NR	TER
Nie2015	randomized control method	4	2/1	None	1 months	VAS, NE,ND,TD
Sun2015	randomized controlled	4	None	NR	NR	TER,NE,TD
Wang2016	random number table	4	1/2	1/2	3 months	TER,VAS,TD,NE,ET-1,CGRP
Yuan2017	randomized controlled	7	None	NR	6 months	TER,VAS,AT,NE
Zhang2017	random number table	4	None	NR	NR	TER
Liu2017	randomized controlled	4	None	NR	1 months	TER, VAS
Wu2017	random number table	4	None	NR	6 months	TER, GMP-140, TXB2, 5-HT, CGRP, ABV
Ma2018	random number table	2	None	4/3	NR	TER,VAS, PSQI
Zuo2018	randomized controlled	6	None	NR	6 months	TER,VAS,NE,AT
Cui2018	random number table	8	None	NR	3 months	TER,5-HT,*β*-EP,SP
Zhao2018	randomized controlled	4	None	NR	1 months	TER,TD,

E, experiment group; C, control group; NR, not report.

IHS, International Headache Society. TCGNCM, The Clinical Guidelines for the New Chinese Medicine. CDSS, Clinical Diagnosis Symptoms of Study. STCMDE, Standard of TCM Diagnosis and Efficacy. PN, Practical Neurology. DCTPIMC, Diagnostic Criteria and Treatment Points of Internal Medical Diseases. HCN, Handbook of Clinical Neurology. IMTCM, Internal Medicine of Traditional Chinese Medicine. GFDTMC, Guidelines for diagnosis and treatment of migraine in China. HMNDT, Handbook of Modern Neurology Diagnosis and Treatment.

PIM, Practical Internal Medicine. TER, Total Efficacy Rate. ABV, average blood velocity in the brain. AT, analgesic time. HT, heal time. SP, systolic pressure. DP, diastolic pressure. SF, seizure frequency.

NE, number of episodes. ND, number of days of attack.

TD, time of duration.

**Table 3:** Meta-analysis of indexes.

Indexes	No. of study	Study	Case E/C	WMD[95%CI]	Z	P
*β*-EP	2	sun2016,cui2018	103	10.16[8.56, 11.76]	12.47	<0.00001
SP	2	sun2016,cui2018	103	-8.84[-10.14,-7.45]	13.30	<0.00001
CGRP	2	Wang2016,wu2017	80	-2.31[-4.01,-0.61]	2.66	<0.00001
GMP-140	2	yuan2012, wu2017	100	-1.22[-2.91,0.48]	1.41	<0.00001
TXB2	2	yuan2012, wu2017	100	-8.36[-16.80,0.08]	1.94	<0.00001
High-cut viscosity	1	yuan2012	46	-2.31[-2.55,-2.07]	18.65	<0.00001
Low-cut viscosity	1	yuan2012	46	-3.91[-4.33,-3.49]	18.24	<0.00001
plasma viscosity	1	yuan2012	46	-0.73[-0.82,-0.64]	15.73	<0.00001
fibrinogen	1	yuan2012	46	-1.43[-1.89,-0.97]	6.13	<0.00001

## Data Availability

All data generated or analyzed during this study are included in this published article.

## Abbreviations

CXCT: Chuanxiong Chatiao san
RCT: Randomized controlled trial
WCM: Western conventional medicine
TER: Total efficiency rate
AE: Adverse event.
